# The impact of physical exercise on primary school teachers’ professional ethics: the mediating role of self-control

**DOI:** 10.3389/fpsyg.2024.1532734

**Published:** 2025-01-15

**Authors:** Chu Kequn, Yang Xiuqing, Zhang Tinghui

**Affiliations:** ^1^College of Educational, Guangxi Science and Technology Normal University, Laibin, China; ^2^Institute for Future Education, Guangxi Science and Technology Normal University, Laibin, China

**Keywords:** physical exercise, self-control, professional ethics, elementary school teachers, mediating effect

## Abstract

**Background:**

Physical exercise has been shown to positively impact psychological and behavioral outcomes, yet its influence on professional ethics in educators remains underexplored. Understanding the mechanisms underlying this relationship is essential for developing interventions to promote ethical behavior in educational contexts.

**Objectives:**

This study aims to investigate the relationship between physical exercise and professional ethics among elementary school teachers, with a specific focus on the mediating role of self-control.

**Methods:**

A sample of 380 elementary school teachers was recruited from Guangxi and Shandong provinces in China. Data on physical exercise, self-control, and professional ethics were collected using validated scales. Structural equation modeling (SEM) was employed to analyze the direct and indirect effects of physical exercise on professional ethics.

**Results:**

The findings revealed a significant positive correlation between physical exercise, self-control, and professional ethics. Self-control was found to partially mediate the relationship, with physical exercise having both a direct positive effect (β = 0.25, *p* < 0.01) and a stronger indirect effect (β = 0.50, *p* < 0.001) on professional ethics through self-control. The indirect effect accounted for 57.3% of the total effect.

**Conclusion:**

This study highlights the dual-pathway mechanism through which physical exercise enhances professional ethics, directly and indirectly via self-control. Practical implications include promoting physical activity and self-regulatory training as strategies to foster ethical behavior in educators. These findings provide a foundation for future research and interventions in educational settings.

## Introduction

Professional ethics in elementary education serves as a cornerstone for shaping students’ development and maintaining societal moral standards. Elementary school teachers’ ethical behavior directly influences students’ value systems and contributes to the broader goal of fostering an ethical society (Sharma, 2024). Professional ethics in teaching encompasses integrity, fairness, and commitment to student welfare. However, the factors influencing these ethical behaviors, particularly the interaction between personal habits and psychological mechanisms, remain underexplored (Bashir et al., 2023). Specifically, how personal habits and psychological mechanisms interact to shape ethical behavior in teaching professionals has yet to be fully understood. This study focuses on the interplay between physical exercise, self-control, and professional ethics, providing new insights into this underexplored area.

Physical exercise exerts profound effects on mental and cognitive functions, which can significantly influence ethical behavior and decision-making (Mandolesi et al., 2018). Within the framework of Self-Determination Theory (SDT), physical exercise satisfies basic psychological needs—competence, autonomy, and relatedness—thereby fostering emotional resilience and cognitive clarity, both of which are essential for moral reasoning and ethical behavior (Deci and Ryan, 2000). As a self-regulated activity, physical exercise aligns with the principles of SDT, promoting intrinsic motivation and sustained ethical engagement. Meanwhile, Social Cognitive Theory (SCT) emphasizes self-control as a vital mechanism for maintaining ethical alignment under challenging circumstances (Bandura, 1991). Teachers with higher self-control are better positioned to regulate impulses, set long-term goals, and navigate ethical dilemmas effectively. This study bridges these theoretical perspectives by exploring how physical exercise and self-control jointly contribute to teachers’ professional ethics.

Empirical studies support the association between physical exercise and ethical behavior(Gao et al., 2018; Ghalenoei et al., 2021; Leese et al., 2022). Research indicates that individuals who regularly engage in physical activity exhibit higher emotional stability and prosocial tendencies, which facilitate ethical decision-making (Van den Bergh, 2021). Similarly, workplace studies suggest that physically active employees adhere more consistently to ethical norms, potentially due to improved self-regulatory capacities stemming from exercise (Hagger and Chatzisarantis, 2010). Self-control, a well-established predictor of ethical behavior, is significantly enhanced by physical exercise. Tangney et al. (2004) found that individuals who exercise regularly demonstrate stronger impulse control and emotional resilience, both of which are integral to ethical alignment in challenging situations.

Many studies have shown that self-control can positively predict professional ethics(Brownstein, 2018; Fauziah and Khadzil, 2023; Sosik et al., 2019). Duckworth et al. (2016) emphasized that self-control enables individuals to resist unethical temptations and consistently act in accordance with moral values. Baumeister and Vohs (2016) further noted that self-control fosters ethical congruence between values and actions, underscoring its importance in maintaining professional ethics. However, while pairwise relationships among physical exercise, self-control, and professional ethics are well-supported, their integrated dynamics remain unclear.

This study makes a unique contribution by addressing the gap in understanding the behavioral mechanisms that link physical exercise to professional ethics, a relationship that remains underexplored in educational research. Existing studies have primarily focused on the cognitive and emotional benefits of physical exercise, but little attention has been given to its potential influence on ethical behavior, particularly among educators. To bridge this gap, this study integrates insights from Self-Determination Theory (SDT) and Social Cognitive Theory (SCT) and proposes that self-control mediates the relationship between physical exercise and professional ethics. Specifically, it hypothesizes that physical exercise enhances professional ethics through two pathways: (1) a direct positive effect and (2) an indirect effect mediated by self-control. This dual-pathway hypothesis provides a novel framework for understanding the interplay between physical and psychological factors in shaping ethical behavior. Moreover, the findings are expected to offer practical strategies for fostering ethical conduct among educators by promoting physical activity and strengthening self-regulatory capacities through targeted interventions.

## Methods

### Participants

This study employed a stratified random sampling method to recruit 400 elementary school teachers from 20 schools across Guangxi and Shandong provinces in China. Stratification was based on school type (urban, suburban, and rural) and teaching experience (novice, intermediate, and experienced) to ensure diversity and representativeness of the sample. This approach was designed to capture variations in demographic and professional characteristics across different educational contexts.

To ensure data quality, clear criteria were established for identifying and excluding invalid questionnaires. A questionnaire was deemed invalid if it contained missing responses for more than 10% of items, exhibited a repetitive response pattern (e.g., identical answers across all Likert-scale items), or showed inconsistencies between related items. Of the 400 distributed questionnaires, 380 valid responses were retained, resulting in a 95% response rate.

Although formal ethical approval was not required under local regulations, the study adhered to international ethical standards to ensure the rights and well-being of participants. Participation was entirely voluntary, and all participants were informed of the study’s purpose, procedures, and their right to withdraw at any time without consequences. Anonymity was maintained by assigning unique identification codes to each questionnaire, and no personal identifiers were collected. Participants were also assured that their responses would be used solely for academic research purposes. These measures align with the ethical principles of confidentiality, informed consent, and voluntary participation.

The sample comprised 62% female (*n* = 236) and 38% male participants (*n* = 144). The age of participants ranged from 24 to 50 years, with an average age of 34.2 years (SD = 6.5). Teaching experience varied from 2 to 25 years, with a mean of 10.3 years (SD = 5.7). Table 1 summarizes the demographic characteristics of the participants, including gender, age, and years of teaching experience.

**Table 1:** Demographic characteristics of the participants.

Demographic Variables	Categories	Numbers
Gender	Male	144
	Female	236
Age	<30 years	96
	30–39 years	204
	≥40 years	80
Teaching Experience	<5 years	72
	5–10 years	142
	>10 years	166

### Measures

#### Physical exercise

Physical exercise levels were assessed using the Chinese version of the International Physical Activity Questionnaire (IPAQ; Craig et al., 2003), which has been widely validated for use in Chinese populations. The Chinese version of the IPAQ was revised and validated by Li et al. (2005), with strong psychometric properties reported in previous studies. In this study, Cronbach’s α for the IPAQ was 0.88, indicating good internal consistency. Participants reported the frequency, duration, and intensity of physical activities over the past month, and the results were calculated as MET-minutes per week. Based on IPAQ guidelines, participants were categorized into low, moderate, or high physical activity levels.

#### Self-control

Self-control was measured using the Chinese version of the Brief Self-Control Scale (BSCS; Tangney et al., 2004; Zhao et al., 2015). The BSCS consists of 13 items across two dimensions: impulse control (e.g., “I am good at resisting temptation”) and goal regulation (e.g., “I am able to work effectively toward long-term goals”). Previous studies have demonstrated strong validity and reliability for this scale in Chinese contexts (de Ridder et al., 2012; Zhao et al., 2015). In this study, the overall Cronbach’s α was 0.92, with α values of 0.90 and 0.88 for the impulse control and goal regulation subscales, respectively, indicating excellent reliability.

#### Professional ethics

Professional ethics were assessed using the Chinese version of the Professional Ethics Scale for Teachers (PEST; Husu and Tirri, 2007; Zhang et al., 2018), which has been specifically validated for Chinese educators. The scale consists of 20 items across three dimensions: integrity, fairness, and responsibility. Sample items include “I treat all students equally regardless of their background.” The scale has demonstrated strong construct validity in previous studies and achieved a Cronbach’s α of 0.94 in this study, with subscale α values of 0.91 (integrity), 0.89 (fairness), and 0.90 (responsibility). These results confirm the scale’s high reliability and suitability for the present research.

### Data analysis

SPSS 23.0 was used for descriptive statistics and correlation analysis to explore the relationships among physical exercise, self-control, and professional ethics. Structural equation modeling (SEM) was conducted using Amos 22.0 to examine the mediating effect of self-control. SEM was chosen for its ability to analyze complex relationships between observed and latent variables, as well as its capacity to simultaneously estimate direct and indirect effects. The bootstrap method with 5,000 resamples was used to test the significance of indirect effects, providing bias-corrected confidence intervals. Model fit indices, including *χ*^2^/df, CFI, NFI, IFI, and RMSEA, were used to assess the adequacy of the model. These methods ensured the robustness and reliability of the mediation analysis.

## Results

### Descriptive statistics and correlations

Descriptive statistics for the three primary variables—physical exercise, self-control, and professional ethics—are presented in Table 2. On average, participants engaged in moderate levels of physical activity (M = 4,560 MET-min/week, SD = 1,243) and exhibited moderately high self-control (M = 3.78, SD = 0.58). Professional ethics scores were also relatively high (M = 4.12, SD = 0.61), indicating that participants demonstrated strong ethical conduct overall. Reliability coefficients for all variables were satisfactory, with Cronbach’s α values ranging from 0.88 to 0.94, confirming the internal consistency of the scales.

**Table 2:** Descriptive statistics and reliability coefficients (*n* = 380).

Variable	M	SD	Cronbach’s α
Physical Exercise	4,560 MET-min/week	1,243	0.88
Self-Control	3.78	0.58	0.92
Impulse Control	3.65	0.62	
Goal Regulation	3.91	0.57	
Professional Ethics	4.12	0.61	0.94
Integrity	4.18	0.63	
Fairness	4.07	0.59	
Responsibility	4.10	0.58	

Correlation analysis revealed significant positive relationships among the primary variables (see Table 3). Physical exercise was moderately correlated with self-control (r = 0.42, *p* < 0.01) and professional ethics (r = 0.36, p < 0.01). Self-control showed a stronger correlation with professional ethics (r = 0.54, *p* < 0.001), highlighting its critical role. Among the dimensions, both impulse control and goal regulation were positively associated with all sub-dimensions of professional ethics, including integrity, fairness, and responsibility. These findings provide preliminary support for the mediating role of self-control in the relationship between physical exercise and professional ethics. These findings provide preliminary support for the mediating role of self-control in the relationship between physical exercise and professional ethics.

**Table 3:** Correlations between variables (*n* = 380, M ± SD).

Variable	1	2	3	4	5	6	7	8
1 Physical Exercise								
2 Self-Control	0.42**							
3 Impulse Control	0.38**	0.38**						
4 Goal Regulation	0.41**	0.49***	0.52***					
5 Professional Ethics	0.36**	0.54***	0.49***	0.50***				
6 Integrity	0.32**	0.52***	0.48***	0.50***	0.71***			
7 Fairness	0.34**	0.49***	0.47***	0.52***	0.75***	0.66***		
8 Responsibility	0.31**	0.50***	0.47***	0.48***	0.68***	0.71***	0.65***	

**p < 0.01,* **p < 0.001.

### Mediating effect of self-control

**Figure 1:**
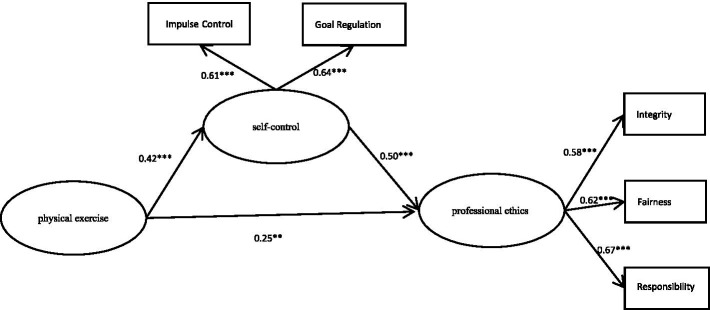
Mediation effect model. **p < 0.01, ***p < 0.001.

The mediating effect of self-control in the relationship between physical exercise and professional ethics was examined using structural equation modeling (SEM). The model demonstrated acceptable fit indices: *χ*^2^ = 215.36, df = 85, *χ*^2^/df = 2.53, NFI = 0.91, CFI = 0.94, IFI = 0.94, RMSEA = 0.05. Results showed that physical exercise had both a significant direct effect on professional ethics (β = 0.25, *p* < 0.01) and an indirect effect mediated by self-control (β = 0.50, *p* < 0.001). The direct effect accounted for 42.70% of the total effect, while the mediated (indirect) effect contributed 57.30%, underscoring the dominant role of self-control in this relationship. These findings confirm that self-control partially mediates the influence of physical exercise on professional ethics and provide strong evidence for its critical role as a psychological mechanism linking these variables. A detailed representation of these relationships is provided in Figure 1. Table 4 provides a summary of the standardized path coefficients and the proportion of variance explained (R^2^) for each variable.

**Table 4:** Path coefficients and variance explained (R^2^).

Path	Standardized Coefficient (β)	*p*-value	R^2^ (Variance Explained)
Physical Exercise → Self-Control	0.42	<0.001	0.18
Self-Control → Professional Ethics	0.50	<0.001	—
Physical Exercise → Professional Ethics	0.25	<0.01	0.35

## Discussion

This study explored the relationships among physical exercise, self-control, and professional ethics, with a particular focus on the mediating role of self-control. The findings provide theoretical and practical insights into how physical and psychological factors interact to influence professional ethics among elementary school teachers.

The findings of this study revealed a significant direct relationship between physical exercise and professional ethics, consistent with prior research emphasizing the psychological and behavioral benefits of regular physical activity. Van den Bergh (2021) highlighted that physical activity enhances prosocial tendencies and emotional stability, both of which are essential for ethical behavior. Similarly, Hagger and Chatzisarantis (2010) demonstrated that improved self-regulatory capacities through physical exercise contribute to better alignment between values and actions, which supports ethical decision-making. These findings suggest that teachers who engage in regular physical exercise may develop stronger resilience and emotional regulation, enabling them to handle ethical challenges in their professional roles more effectively. Furthermore, the partial mediating role of self-control identified in this study aligns with previous work by Tangney et al. (2004), who emphasized self-control as a critical capacity for resisting unethical temptations and maintaining moral integrity. By integrating these insights, this study reinforces the theoretical and practical importance of promoting physical activity and self-regulatory skills in educational settings to foster ethical conduct among teachers.

Self-control emerged as a significant mediator in the relationship between physical exercise and professional ethics, providing a deeper understanding of the underlying mechanisms. Physical exercise may enhance self-control by promoting emotional resilience and cognitive clarity, as suggested by prior research (Van den Bergh, 2021). Regular physical activity has been shown to reduce stress levels and improve executive functioning, both of which are critical for impulse regulation and long-term goal alignment (Mandolesi et al., 2018). These improvements enable individuals to navigate ethical dilemmas more effectively by aligning their actions with moral values. This finding aligns with the principles of Self-Determination Theory (SDT), which posits that physical activity satisfies basic psychological needs such as competence and autonomy, thereby fostering intrinsic motivation and self-regulation. Additionally, Social Cognitive Theory (SCT) emphasizes that self-control serves as a vital capacity for resisting unethical temptations and adhering to professional standards under pressure (Bandura, 1991). By integrating these theoretical perspectives, this study highlights self-control as a pivotal mechanism linking physical and psychological factors to ethical decision-making. These results align with prior studies showing that physical exercise improves self-regulatory capacities, including impulse control and goal regulation (Tangney et al., 2004; Hagger and Chatzisarantis, 2010), which in turn facilitate ethical alignment between values and actions (Baumeister and Vohs, 2016).

The findings of this study offer actionable strategies for fostering ethical behavior among educators. First, promoting physical exercise as part of professional development programs can serve as a dual-purpose intervention, enhancing both physical health and ethical decision-making. Schools and educational institutions could implement structured physical activity initiatives, such as regular exercise classes, team sports, or workplace wellness programs, to support teachers’ well-being and professional ethics. These programs should be designed to accommodate teachers’ busy schedules and emphasize consistency to maximize long-term benefits.

Second, interventions aimed at strengthening self-control should be incorporated into teacher training programs. Mindfulness training, for example, can help teachers develop emotional regulation and impulse control, which are critical for navigating ethical dilemmas. Similarly, workshops focused on goal-setting and self-monitoring can enhance teachers’ ability to align their actions with professional standards and moral values. By combining physical activity with self-control development, these strategies offer a comprehensive approach to fostering ethical behavior in educational contexts.

## Limitations and future directions

Despite its contributions, this study has several limitations that should be addressed in future research. First, the cross-sectional design prevents the establishment of causal relationships among physical exercise, self-control, and professional ethics. Longitudinal studies and experimental designs, such as randomized controlled trials, are necessary to confirm the temporal order and causal pathways proposed in this study.

Second, the reliance on self-reported measures may introduce response biases, such as social desirability or overestimation of behaviors like physical activity and ethical conduct. To mitigate this limitation, future research should incorporate objective measures, such as wearable fitness trackers for physical activity or third-party assessments of professional ethics, to enhance data reliability and accuracy.

Third, the sample was limited to elementary school teachers in specific regions of China, which may restrict the generalizability of the findings to other professional groups or cultural contexts. Cross-cultural analyses involving diverse teacher populations across different countries and educational systems would enhance the external validity of the results and provide valuable insights into how cultural norms influence ethical behavior.

Finally, potential confounding variables, such as emotional intelligence, workplace stress, or job satisfaction, were not controlled for in this study. Future research should explore these additional factors as mediators or moderators to provide a more nuanced understanding of how physical and psychological resources interact to shape ethical behavior. By addressing these limitations and research directions, future studies can contribute to the development of evidence-based strategies for fostering ethical behavior in educational and other professional settings.

## Conclusion

This study underscores the pivotal role of self-control as a mediating mechanism in the relationship between physical exercise and professional ethics. The findings reveal that physical exercise not only directly enhances professional ethics but also exerts a stronger indirect influence through the mediating effect of self-control. This dual-pathway mechanism provides a comprehensive framework for understanding how physical and psychological factors interact to shape ethical behavior.

From a practical perspective, the results highlight the importance of promoting physical activity and self-regulatory skills among educators. Schools and educational institutions should consider integrating structured physical activity programs and self-control training into professional development initiatives to foster ethical decision-making. Such interventions could include mindfulness training, goal-setting workshops, or workplace wellness programs designed to enhance emotional resilience, impulse control, and moral reasoning.

By bridging theoretical insights with practical applications, this study contributes to the growing body of research on ethical behavior in education and provides a foundation for future investigations. These findings pave the way for more targeted and evidence-based strategies to promote professional ethics in educational and other professional contexts.

## Data Availability

The raw data supporting the conclusions of this article will be made available by the authors, without undue reservation.
